# Effectiveness of a Third Dose of Pfizer-BioNTech and Moderna Vaccines in Preventing COVID-19 Hospitalization Among Immunocompetent and Immunocompromised Adults — United States, August–December 2021

**DOI:** 10.15585/mmwr.mm7104a2

**Published:** 2022-01-28

**Authors:** Mark W. Tenforde, Manish M. Patel, Manjusha Gaglani, Adit A. Ginde, David J. Douin, H. Keipp Talbot, Jonathan D. Casey, Nicholas M. Mohr, Anne Zepeski, Tresa McNeal, Shekhar Ghamande, Kevin W. Gibbs, D. Clark Files, David N. Hager, Arber Shehu, Matthew E. Prekker, Heidi L. Erickson, Michelle N. Gong, Amira Mohamed, Nicholas J. Johnson, Vasisht Srinivasan, Jay S. Steingrub, Ithan D. Peltan, Samuel M. Brown, Emily T. Martin, Arnold S. Monto, Akram Khan, Catherine L. Hough, Laurence W. Busse, Abhijit Duggal, Jennifer G. Wilson, Nida Qadir, Steven Y. Chang, Christopher Mallow, Carolina Rivas, Hilary M. Babcock, Jennie H. Kwon, Matthew C. Exline, Mena Botros, Adam S. Lauring, Nathan I. Shapiro, Natasha Halasa, James D. Chappell, Carlos G. Grijalva, Todd W. Rice, Ian D. Jones, William B. Stubblefield, Adrienne Baughman, Kelsey N. Womack, Jillian P. Rhoads, Christopher J. Lindsell, Kimberly W. Hart, Yuwei Zhu, Eric A. Naioti, Katherine Adams, Nathaniel M. Lewis, Diya Surie, Meredith L. McMorrow, Wesley H. Self, IVY Network, IVY Network, Nicole Calhoun, Kempapura Murthy, Judy Herrick, Amanda McKillop, Eric Hoffman, Martha Zayed, Michael Smith, Ryan Kindle, Lori-Ann Kozikowski, Lesley De Souza, Scott Ouellette, Sherell Thornton-Thompson, Omar Mehkri, Kiran Ashok, Susan Gole, Alexander King, Bryan Poynter, Caitlin ten Lohuis, Nicholas Stanley, Audrey Hendrickson, Sean Caspers, Walker Tordsen, Olivia Kaus, Tyler Scharber, Jeffrey Jorgensen, Robert Bowers, Jennifer King, Valerie Aston, Richard E. Rothman, Harith Ali, Rahul Nair, Sarah Karow, Emily Robart, Paulo Nunes Maldonado, Maryiam Khan, Preston So, Olivia Krol, Jesus Martinez, Zachary Zouyed, Michael Acosta, Reihaneh Bazyarboroujeni, Haeun Jung, Raju Reddy, Richard Zhang, Alexandra Jun Gordon, Joe Levitt, Cynthia Perez, Anita Visweswaran, Jonasel Roque, Sukantha Chandrasekaran, Los Angeles, Trevor Frankel, Los Angeles, Omai Garner, Los Angeles, Jennifer Goff, David Huynh, Kelly Jensen, Conner Driver, Michael Carricato, Ian Chambers, Paul Nassar, Lori Stout, Zita Sibenaller, Alicia Walter, Jasmine Mares, Spenser Pfannenstiel, Hayley Gershengorn, EJ McSpadden, Rachel Truscon, Lara Thomas, Ramsay Bielak, Weronika Damek Valvano, Rebecca Fong, William J. Fitzsimmons, Christopher Blair, Julie Gilbert, Christine D. Crider, Kyle A. Steinbock, Thomas C. Paulson, Layla A. Anderson, Christy Kampe, Jakea Johnson, Laura L. Short, Lauren J. Ezzell, Margaret E. Whitsett, Rendie E. McHenry, Samarian J. Hargrave, Marica Blair, Jennifer L. Luther, Claudia Guevara Pulido, Bryan P. M. Peterson, Mary LaRose, Leigha Landreth, Madeline Hicks, Lisa Parks, Jahnavi Bongu, David McDonald, Candice Cass, Sondra Seiler, David Park, Tiffany Hink, Meghan Wallace, Carey-Ann Burnham, Olivia G. Arter

**Affiliations:** ^1^CDC COVID-19 Emergency Response Team; ^2^Baylor Scott & White Health, Temple, Texas; ^3^Texas A&M University College of Medicine, Temple, Texas; ^4^University of Colorado School of Medicine, Aurora, Colorado; ^5^Vanderbilt University Medical Center, Nashville, Tennessee; ^6^University of Iowa, Iowa City, Iowa; ^7^Wake Forest University Baptist Medical Center, Winston-Salem, North Carolina; ^8^The Johns Hopkins Hospital, Baltimore, Maryland; ^9^Hennepin County Medical Center, Minneapolis, Minnesota; ^10^Montefiore Medical Center, Albert Einstein College of Medicine, New York City borough of the Bronx, New York; ^11^University of Washington School of Medicine, Seattle, Washington; ^12^Baystate Medical Center, Springfield, Massachusetts; ^13^Intermountain Medical Center and University of Utah, Salt Lake City, Utah; ^14^University of Michigan School of Public Health, Ann Arbor, Michigan; ^15^Oregon Health & Science University Hospital, Portland, Oregon; ^16^Emory University School of Medicine, Atlanta, Georgia; ^17^Cleveland Clinic, Cleveland, Ohio; ^18^Stanford University School of Medicine, Palo Alto, California; ^19^Ronald Reagan-UCLA Medical Center, Los Angeles, California; ^20^University of Miami, Miami, Florida; ^21^Washington University, St. Louis, Missouri; ^22^Ohio State University Wexner Medical Center, Columbus, Ohio; ^23^University of Michigan School of Medicine, Ann Arbor, Michigan; ^24^Beth Israel Deaconess Medical Center, Boston, Massachusetts.; Baylor Scott & White Health; Baylor Scott & White Health; Baylor Scott & White Health; Baylor Scott & White Health; Baylor Scott & White Health; Baylor Scott & White Health; Baylor Scott & White Health; Baystate Medical Center; Baystate Medical Center; Baystate Medical Center; Baystate Medical Center; Baystate Medical Center; Cleveland Clinic; Cleveland Clinic; Cleveland Clinic; Cleveland Clinic; Cleveland Clinic; Emory University; Emory University; Hennepin County Medical Center; Hennepin County Medical Center; Hennepin County Medical Center; Hennepin County Medical Center; Hennepin County Medical Center; Intermountain Medical Center; Intermountain Medical Center; Intermountain Medical Center; Intermountain Medical Center; Johns Hopkins University; Johns Hopkins University; Montefiore Medical Center; Jen-Ting (Tina) Chen; Montefiore Medical Center; Ohio State University; Ohio State University; Ohio State University; Ohio State University; Ohio State University; Oregon Health & Science University; Oregon Health & Science University; Oregon Health & Science University; Oregon Health & Science University; Oregon Health & Science University; Oregon Health & Science University; Oregon & Health Science University; Oregon Health & Science University; Stanford University; Stanford University; Stanford University; Stanford University; Stanford University; University of California; University of California; University of California; UCHealth University of Colorado Hospital; UCHealth University of Colorado Hospital; UCHealth University of Colorado Hospital; UCHealth University of Colorado Hospital; UCHealth University of Colorado Hospital; UCHealth University of Colorado Hospital; University of Iowa; University of Iowa; University of Iowa; University of Iowa; University of Iowa; University of Iowa; University of Miami; University of Michigan; University of Michigan; University of Michigan; University of Michigan; University of Michigan; University of Michigan; University of Michigan; University of Michigan; University of Michigan; University of Washington; University of Washington; University of Washington; University of Washington; Vanderbilt University Medical Center; Vanderbilt University Medical Center; Vanderbilt University Medical Center; Vanderbilt University Medical Center; Vanderbilt University Medical Center; Vanderbilt University Medical Center; Vanderbilt University Medical Center; Vanderbilt University Medical Center; Vanderbilt University Medical Center; Vanderbilt University Medical Center; Vanderbilt University Medical Center; Wake Forest University; Wake Forest University; Wake Forest University; Wake Forest University; Washington University; Washington University; Washington University; Washington University; Washington University; Washington University; Washington University; Washington University; Washington University.

COVID-19 mRNA vaccines (BNT162b2 [Pfizer-BioNTech] and mRNA-1273 [Moderna]) provide protection against infection with SARS-CoV-2, the virus that causes COVID-19, and are highly effective against COVID-19–associated hospitalization among eligible persons who receive 2 doses (*1*,*2*). However, vaccine effectiveness (VE) among persons with immunocompromising conditions* is lower than that among immunocompetent persons (*2*), and VE declines after several months among all persons (*3*). On August 12, 2021, the Food and Drug Administration (FDA) issued an emergency use authorization (EUA) for a third mRNA vaccine dose as part of a primary series ≥28 days after dose 2 for persons aged ≥12 years with immunocompromising conditions, and, on November 19, 2021, as a booster dose for all adults aged ≥18 years at least 6 months after dose 2, changed to ≥5 months after dose 2 on January 3, 2022 (*4*,*5**,**6*). Among 2,952 adults (including 1,385 COVID-19 case-patients and 1,567 COVID-19–negative controls) hospitalized at 21 U.S. hospitals during August 19–December 15, 2021, effectiveness of mRNA vaccines against COVID-19–associated hospitalization was compared between adults eligible for but who had not received a third vaccine dose (1,251) and vaccine-eligible adults who received a third dose ≥7 days before illness onset (312). Among 1,875 adults without immunocompromising conditions (including 1,065 [57%] unvaccinated, 679 [36%] 2-dose recipients, and 131 [7%] 3-dose [booster] recipients), VE against COVID-19 hospitalization was higher among those who received a booster dose (97%; 95% CI = 95%–99%) compared with that among 2-dose recipients (82%; 95% CI = 77%–86%) (p <0.001). Among 1,077 adults with immunocompromising conditions (including 324 [30%] unvaccinated, 572 [53%] 2-dose recipients, and 181 [17%] 3-dose recipients), VE was higher among those who received a third dose to complete a primary series (88%; 95% CI = 81%–93%) compared with 2-dose recipients (69%; 95% CI = 57%–78%) (p <0.001). Administration of a third COVID-19 mRNA vaccine dose as part of a primary series among immunocompromised adults, or as a booster dose among immunocompetent adults, provides improved protection against COVID-19–associated hospitalization.

During August 19–December 15, 2021, a period in which the SARS-CoV-2 B.1.617.2 (Delta) variant was predominant, adults admitted to 21 hospitals in 18 states within the Influenza or Other Viruses in the Acutely Ill Network (IVY Network) who received testing for SARS-CoV-2 were included in a VE analysis. The analysis start date of August 19, 2021, was one week after the EUA of a third dose for persons with immunocompromising conditions. VE against COVID-19 hospitalization was compared between two groups: 1) adults who had completed a 2-dose mRNA vaccination series and were eligible for but had not received a third dose^†^; and 2) adults who were eligible for and had received a third dose ≥7 days before illness onset. VE was calculated for 2-dose and 3-dose recipients by comparing odds of antecedent vaccination between COVID-19 case-patients and control patients who did not have COVID-19. Case-patients had COVID-19–like illness^§^ and received positive SARS-CoV-2 test results by a nucleic acid amplification test (NAAT) or antigen test within 10 days of illness onset. Control patients were hospitalized with or without COVID-19–like illness and received negative SARS-CoV-2 test results by NAAT.

Patients or their proxies were interviewed regarding patient demographic and clinical characteristics, and medical record searches were completed to collect information about chronic medical conditions. Information about receipt of prior COVID-19 vaccination doses, including dates, locations, and vaccine product received, was obtained through self-report and review of source documentation (including state vaccination registries, medical records, and vaccination cards). A patient was considered to be vaccinated if vaccination could be verified through source documentation or by self-report, including dates and location. Three vaccination groups were considered: 1) unvaccinated patients, who had received no COVID-19 vaccine doses before illness onset; 2) 2-dose mRNA vaccine recipients, who were eligible for but had not received a third vaccine dose or had received a third dose <7 days before illness onset; and 3) 3-dose mRNA vaccine recipients, who received a third dose ≥7 days before illness onset. Patients were excluded if they were admitted to the hospital ≤7 days after EUA authorization of a third dose, received one or more vaccine doses but did not qualify for inclusion in the 2-dose or 3-dose vaccine group,^¶^ received a non-mRNA vaccine (e.g., Ad26.COV2.S [Janssen (Johnson & Johnson)]), or if verification of vaccination was pending.

VE against COVID-19–associated hospitalization was estimated using logistic regression, comparing the odds of being vaccinated versus being unvaccinated among case-patients and controls using the equation VE = 100 × (1 − adjusted odds ratio). The regression model included case-patient or control status as the outcome variable and an indicator variable for vaccination group (unvaccinated, 2-dose recipient, or 3-dose recipient), and was adjusted for admission date, region of hospital (U.S. Department of Health and Human Services or U.S. Census Bureau), age group (18–49, 50–64, or ≥65 years), sex, and self-reported race and ethnicity. Separate models were generated for immunocompetent adults and adults with immunocompromising conditions. To compare VE among 2-dose versus 3-dose mRNA vaccine recipients, post hoc comparisons were performed with the pwcompare function in Stata with a two-sided significance threshold of p<0.05. Analyses were conducted using Stata software (version 16.0; StataCorp). This activity was determined to be public health surveillance by each participating site and CDC and was conducted consistent with applicable federal law and CDC policy.**

During August 19–December 15, 2021, the IVY Network enrolled 4,094 adults aged ≥18 years. After excluding 1,142 patients (619 because they did not belong to an included vaccination group, 386 because the patients had received a non-mRNA vaccine or vaccination verification was incomplete, and 137 because they met other exclusion criteria), 2,952 hospitalized patients were included (1,385 case-patients and 1,567 non–COVID-19 controls). Among all participants, median age was 62 years, 49% of patients were female, 58% were non-Hispanic White, and 36% had an immunocompromising condition. Among the 1,385 case-patients, 931 (67%), 408 (29%), and 46 (3%) had received 0, 2, and 3 mRNA vaccine doses, respectively. Among 1,567 non–COVID-19 controls, 458 (29%), 843 (54%), and 266 (17%) had received 0, 2, and 3 mRNA vaccine doses, respectively. Among patients without immunocompromising conditions (Table 1), 2- and 3-dose recipients were similar in terms of age (median = 69 and 72 years, respectively) but differed in self-reported race/ethnicity distribution (a higher percentage of non-Hispanic White persons were among 3-dose recipients; p = 0.008) and U.S. Census Bureau region of the admitting hospital (p = 0.030). Two-dose recipients were less likely to report working in health care settings (5%) than were 3-dose recipients (10%) (p = 0.023) and were more likely to be enrolled as a COVID-19 case-patient (31%) than were 3-dose recipients (8%) (p<0.001). Among patients with immunocompromising conditions (Table 2), 2-dose recipients were more likely to be enrolled as a case-patient (34%) than were 3-dose recipients (20%) (p<0.001). VE against COVID-19 hospitalization among adults without immunocompromising conditions was 82% (95% CI = 77%–86%) for 2 doses and 97% (95% CI = 95%–99%) for 3 doses (p<0.001) (Table 3). VE against COVID-19 hospitalization among adults with immunocompromising conditions was 69% (95% CI = 57%–78%) for 2 doses and 88% (95% CI = 81%–93%) for 3 doses (p<0.001). In a sensitivity analysis among patients with moderately to severely immunocompromising conditions defined by CDC^††^ (*7*), VE was 65% (95% CI = 49%–76%) for receipt of 2 doses, and 87% (95% CI = 78%–92%) for receipt of 3 doses (p<0.001).

**TABLE 1:** Characteristics of vaccine effectiveness analysis participants without immunocompromising conditions, including case-patients hospitalized with COVID-19 and controls hospitalized without COVID-19, by mRNA vaccination group — 21 hospitals,* 18 U.S. states, August–December 2021

Characteristic	Vaccination group, no./Total no. (%)^†^			P-value for comparison of 2-dose versus 3-dose group^¶^
	Unvaccinated (n = 1,065)	2 mRNA vaccine doses (n = 679)^†^	3 mRNA vaccine doses^§^ (n = 131)	
**COVID-19 case-patients**	744/1,065 (70)	212/679 (31)	10/131 (8)	<0.001
**Age group, yrs**				
18–49	454/1,065 (43)	64/679 (9)	9/131 (7)	0.31
50–64	327/1,065 (31)	179/679 (26)	29/131 (22)	
≥65	284/1,065 (27)	436/679 (64)	93/131 (71)	
**Female sex**	486/1,065 (46)	329/679 (48)	67/131 (51)	0.57
Race/Ethnicity**				
White, non-Hispanic	566/1,065 (53)	409/679 (60)	100/131 (76)	0.008
Black, non-Hispanic	220/1,065 (21)	134/679 (20)	16/131 (12)	
Any race, Hispanic	195/1,065 (18)	85/679 (13)	10/131 (8)	
Non-Hispanic, all other races	57/1,065 (5)	37/679 (5)	2/131 (2)	
Unknown	27/1,065 (3)	14/679 (2)	3/131 (2)	
**U.S. Census region**				
Northeast	176/1,065 (17)	160/679 (24)	20/131 (15)	0.030
Midwest	215/1,065 (20)	151/679 (22)	32/131 (24)	
South	391/1,065 (37)	210/679 (31)	35/131 (27)	
West	283/1,065 (27)	158/679 (23)	44/131 (34)	
**LTCF resident^††^**	25/1,004 (2)	71/663 (11)	17/120 (14)	0.27
**Employed**	343/813 (42)	104/556 (19)	23/96 (24)	0.23
Health care worker	21/813 (3)	26/556 (5)	10/96 (10)	0.023
**Reported ≥1 previous hospitalization in the last year**	267/947 (28)	298/598 (50)	50/119 (42)	0.12
**No. of chronic medical condition types (IQR)**	1 (0–2)	2 (1–3)	2 (1–3)	0.10
**Self-reported previous laboratory-confirmed SARS-CoV-2 infection**	68/1,063 (6)	36/679 (5)	7/131 (5)	0.98
**Vaccine product received**				
Pfizer-BioNTech	NA	386/679 (57)	93/131 (71)	0.001
Moderna	NA	288/679 (42)	35/131 (27)	
Both products	NA	5/679 (0.7)	3/131 (2)	
**Interval between second vaccine dose and illness onset, days, median, (IQR)**	NA	225 (203–248)	257 (240–276)	<0.001
**Interval between second and third vaccine dose, days, median (IQR)**	NA	NA	230 (211–248)	NA
**Interval between third vaccine dose and illness onset, days, median (IQR)**	NA	NA	25 (13–36)	NA

**Abbreviations**: LTCF = long-term care facility; NA = not applicable.

* Hospitals by U.S. Census region included *Northeast*: Baystate Medical Center (Springfield, Massachusetts), Beth Israel Deaconess Medical Center (Boston, Massachusetts), Montefiore Medical Center (New York City borough of the Bronx, New York); *Midwest*: University of Iowa Hospitals & Clinics (Iowa City, Iowa), University of Michigan Hospital (Ann Arbor, Michigan), Hennepin County Medical Center (Minneapolis, Minnesota), Barnes-Jewish Hospital (St. Louis, Missouri), Cleveland Clinic (Cleveland, Ohio), The Ohio State University Wexner Medical Center (Columbus, Ohio); *South*: Vanderbilt University Medical Center (Nashville, Tennessee), University of Miami Medical Center (Miami, Florida), Emory University Hospital (Atlanta, Georgia), The Johns Hopkins Hospital (Baltimore, Maryland), Atrium Health Wake Forest Baptist Medical Center (Winston-Salem, North Carolina), Baylor Scott & White Medical Center (Temple, Texas); *West*: Stanford University Medical Center (Palo Alto, California), Ronald Reagan UCLA Medical Center (Los Angeles, California), UCHealth University of Colorado Hospital (Aurora, Colorado), Oregon Health & Science University Hospital (Portland, Oregon), Intermountain Medical Center (Murray, Utah), University of Washington Medical Center (Seattle, Washington).

† Three vaccination groups were included; unvaccinated patients received no COVID-19 vaccine doses before the date of current illness onset; 2-dose recipients received 2 doses of an mRNA COVID-19 vaccine with the second dose received ≥180 days before the date of illness onset; 3-dose recipients received a third (booster) dose of vaccine with ≥180 days between the second and third dose and ≥7 days between the date of dose 3 receipt and illness onset. Among those who received a third dose with mRNA-1273 from Moderna, one half a dose (0.25 mL) was recommended for the third dose.

**^§^** Dose 3 received ≥6 months after dose 2.

^¶^ Comparisons were made between 2-dose (primary series only) and 3-dose recipients (booster) groups using Pearson's chi-square testing for categorical variables and the non-parametric Wilcoxon rank-sum test for continuous variables.

** Race and ethnic groups self-reported.

^††^ LTCFs included residence in a nursing home, assisted living home, or rehab hospital or other subacute or chronic facility before the hospital admission.

**TABLE 2:** Characteristics of vaccine effectiveness analysis participants with immunocompromising conditions,* including case-patients hospitalized with COVID-19 and controls hospitalized without COVID-19, by mRNA vaccination group – 21 hospitals,^†^ 18 U.S. states, August–December 2021

Characteristic	Vaccination group, no./Total no. (%)^§^			P-value for comparison of 2-dose versus 3-dose group**
	Unvaccinated (n = 324)	mRNA vaccine, 2 doses (n = 572)^†^	mRNA vaccine, 3 doses^¶^; (n = 181)	
**COVID-19 case-patients**	187/324 (58)	196/572 (34)	36/181 (20)	<0.001
**Age group, yrs**				
18–49	100/324 (31)	84/572 (15)	20/181 (11)	0.17
50–64	131/324 (40)	197/572 (34)	55/181 (30)	
≥65	93/324 (29)	291/572 (51)	106/181 (59)	
**Female sex**	168/324 (52)	303/572 (53)	81/181 (45)	0.054
**Race/Ethnicity^††^**				
White, non-Hispanic	176/324 (54)	329/572 (58)	133/181 (73)	0.001
Black, non-Hispanic	82/324 (25)	113/572 (20)	24/181 (13)	
Any race, Hispanic	55/324 (17)	97/572 (17)	14/181 (8)	
Non-Hispanic, all other races	8/324 (2)	25/572 (4)	9/181 (5)	
Unknown	3/324 (0.9)	8/572 (1)	1/181 (0.6)	
**U.S. Census region**				
Northeast	48/324 (15)	94/572 (16)	24/181 (13)	0.56
Midwest	72/324 (22)	131/572 (23)	48/181 (27)	
South	145/324 (45)	206/572 (36)	61/181 (34)	
West	59/324 (18)	141/572 (25)	48/181 (27)	
**LTCF resident^§§^**	8/316 (3)	18/558 (3)	3/178 (2)	0.28
**Employed**	62/258 (24)	108/497 (22)	34/159 (21)	0.93
Health care worker	9/258 (3)	13/497 (3)	5/159 (3)	0.72
**Reported ≥1 previous hospitalization in the last year**	153/307 (50)	317/540 (59)	96/164 (59)	0.97
**No. of chronic medical condition types (IQR)**	3 (2–4)	3 (2–4)	3 (3–4)	0.88
**Self-reported previous laboratory-confirmed SARS-CoV-2 infection**	33/324 (10)	38/572 (7)	12/181 (7)	1.00
**Vaccine product received**				
Pfizer-BioNTech	NA	334/572 (58)	120/181 (66)	<0.001
Moderna	NA	238/572 (42)	57/181 (31)	
Both products	NA	0/572 (—)	4/181 (2)	
**Interval between second vaccine dose and illness onset, days, median (IQR)**	NA	178 (142.5–213)	226 (199–251)	<0.001
**Interval between second and third vaccine dose, days, median (IQR)**	NA	NA	178 (148–202)	NA
**Interval between third vaccine dose and illness onset, days, median (IQR)**	NA	NA	40 (21–68)	NA

**Abbreviations:** LTCF = long-term care facility; NA = not applicable.

* Immunocompromising conditions included having one or more of the following: active solid organ cancer (active cancer defined as treatment for the cancer or newly diagnosed cancer in the past 6 months); active hematologic cancer (such as leukemia, lymphoma, or myeloma); HIV infection without AIDS; AIDS; congenital immunodeficiency syndrome; prior splenectomy; prior solid organ, stem cell, or bone marrow transplant; immunosuppressive medication; systemic lupus erythematosus; rheumatoid arthritis; psoriasis; scleroderma; or inflammatory bowel disease, including Crohn’s disease or ulcerative colitis. Among 2-dose vaccine recipients with immunocompromising conditions (572), 274 (48%) had active cancer, 156 (27%) were on immunosuppressive medications, 118 (21%) had a history of solid organ transplant, and 146 (26%) had another immunocompromising condition; among 3-dose vaccine recipients with immunocompromising conditions (181), 88 (49%) had active cancer, 74 (41%) were on immunosuppressive medications, 65 (36%) had a history of solid organ transplant, and 20 (11%) had another immunocompromising condition.

^†^ Hospitals by U.S. Census region included *Northeast*: Baystate Medical Center (Springfield, Massachusetts), Beth Israel Deaconess Medical Center (Boston, Massachusetts), Montefiore Medical Center (New York City borough of the Bronx, New York); *Midwest*: University of Iowa Hospitals & Clinics (Iowa City, Iowa), University of Michigan Hospital (Ann Arbor, Michigan), Hennepin County Medical Center (Minneapolis, Minnesota), Barnes-Jewish Hospital (St. Louis, Missouri), Cleveland Clinic (Cleveland, Ohio), The Ohio State University Wexner Medical Center (Columbus, Ohio); *South*: Vanderbilt University Medical Center (Nashville, Tennessee), University of Miami Medical Center (Miami, Florida), Emory University Hospital (Atlanta, Georgia), The Johns Hopkins Hospital (Baltimore, Maryland), Atrium Health Wake Forest Baptist Medical Center (Winston-Salem, North Carolina), Baylor Scott & White Medical Center (Temple, Texas); *West*: Stanford University Medical Center (Palo Alto, California), Ronald Reagan UCLA Medical Center (Los Angeles, California), UCHealth University of Colorado Hospital (Aurora, Colorado), Oregon Health & Science University Hospital (Portland, Oregon), Intermountain Medical Center (Murray, Utah), University of Washington Medical Center (Seattle, Washington).

**^§^** Three vaccination groups were included; unvaccinated patients received no COVID-19 vaccine doses before the date of current illness onset; 2-dose recipients received 2 doses of an mRNA COVID-19 vaccine with the second dose received ≥28 days before the date of illness onset; 3-dose recipients received 3 doses of vaccine to complete a primary vaccine series with ≥28 days between the second and third dose and ≥7 days between the date of dose 3 receipt and illness onset.

^¶^ Dose 3 received ≥28 days after dose 2.

** Comparisons were made between 2-dose and 3-dose recipient groups using Pearson's chi-square testing for categorical variables and the nonparametric Wilcoxon rank-sum test for continuous variables.

**^††^** Race and ethnic groups self-reported.

**^§§^** LTCFs included residence in a nursing home, assisted living home, or rehab hospital or other subacute or chronic facility before the hospital admission.

**TABLE 3:** Effectiveness of 2-dose and 3-dose regimens of COVID-19 mRNA vaccines against COVID-19 hospitalization among adults with and without immunocompromising conditions — 21 hospitals, 18 U.S. states,*^,^^†^ August–December 2021

Subgroup	Vaccinated versus unvaccinated, 2 doses		Vaccinated versus unvaccinated, 3 doses		P-value for VE comparison for 2-dose versus 3-dose recipients^§^
	No. vaccinated/Total no. (%)	VE (95% CI)*	No. vaccinated/Total no. (%)	VE (95% CI)*	
**Patients without immunocompromising conditions**					
COVID-19 case-patients	212/956 (22)	82 (77–86)	10/754 (1)	97 (95–99)	<0.001
Control patients	467/788 (59)		121/442 (27)		
**Patients with immunocompromising conditions**					
COVID-19 case-patients	196/383 (51)	69 (57–78)	36/223 (16)	88 (81–93)	<0.001
Control patients	376/513 (73)		145/282 (51)		

**Abbreviation:** VE = vaccine effectiveness.

* VE was estimated using logistic regression, comparing the odds of being vaccinated with the Moderna or Pfizer-BioNTech COVID-19 vaccine product versus being unvaccinated among case-patients and controls using the equation VE = 100 × (1 – adjusted odds ratio). The regression model included three categories for vaccination status: unvaccinated, vaccinated with 2 doses of an mRNA vaccine, or vaccinated with 3 doses of an mRNA vaccine. VE was calculated separately comparing 2-dose recipients to unvaccinated controls and 3-dose recipients to unvaccinated controls. Models were adjusted for date of hospital admission (biweekly intervals), U.S. Department of Health and Human Services region of hospital, age group (18–49, 50–64, or ≥65 years), sex, and race/ethnicity (non-Hispanic White, non-Hispanic Black, Hispanic of any race, non-Hispanic Other, or unknown).

† Hospitals by U.S. Department of Health and Human Services region included: *Region 1*: Baystate Medical Center (Springfield, Massachusetts), Beth Israel Deaconess Medical Center (Boston, Massachusetts); *Region 2*: Montefiore Medical Center (New York City borough of the Bronx, New York); *Region 3*: The Johns Hopkins Hospital (Baltimore, Maryland); *Region 4*: Vanderbilt University Medical Center (Nashville, Tennessee), University of Miami Medical Center (Miami, Florida), Emory University Hospital (Atlanta, Georgia), Atrium Health Wake Forest Baptist Medical Center (Winston-Salem, North Carolina); *Region 5*: University of Michigan Hospital (Ann Arbor, Michigan), Hennepin County Medical Center (Minneapolis, Minnesota), Cleveland Clinic (Cleveland, Ohio), The Ohio State University Wexner Medical Center (Columbus, Ohio); *Region 6*: Baylor Scott & White Medical Center (Temple, Texas); *Region 7*: University of Iowa Hospitals & Clinics (Iowa City, Iowa), Barnes Jewish Hospital (St. Louis, Missouri); *Region 8*: UCHealth University of Colorado Hospital (Aurora, Colorado), Intermountain Medical Center (Murray, Utah); *Region 9*: Stanford University Medical Center (Palo Alto, California), Ronald Reagan UCLA Medical Center (Los Angeles, California); *Region 10*: Oregon Health & Science University Hospital (Portland, Oregon), University of Washington Medical Center (Seattle, Washington).

^§^ Post-hoc pairwise comparisons of VE for 2-dose versus 3-dose vaccine recipients was evaluated using the pwcompare function in Stata software (version 16; StataCorp); a two sided p-value <0.05 indicated a significant difference in VE between groups.

## Discussion

In a multistate network, adults vaccinated with 2 or 3 doses of a COVID-19 mRNA vaccine were protected against COVID-19–associated hospitalization. Significantly higher VE was observed in adults who received a third mRNA vaccine dose either as part of a primary vaccine series (immunocompromised persons) or as a booster dose (immunocompetent persons) compared with those who had received 2 doses. These findings underscore the importance of immunocompromised adults obtaining a third mRNA vaccine dose ≥28 days after the second vaccine dose and of immunocompetent adults receiving a third (booster) dose currently recommended ≥5 months after the second dose.^§§^

This study was conducted during a period of SARS-CoV-2 Delta variant predominance (*8*). VE against the B.1.1.529 (Omicron) variant of SARS-CoV-2 might be lower than for other variants that have circulated widely, possibly because of immune evasion. Early evidence suggests that a third mRNA vaccine dose elicits markedly stronger neutralizing antibody responses to the Omicron variant compared with responses to 2 vaccine doses (*9*), and increases VE against severe disease following infection with the Omicron variant (*10*). The effectiveness of 3 doses of COVID-19 mRNA vaccines against a range of disease severity associated with the Omicron variant needs to be carefully evaluated in different populations.

The findings in this report are subject to at least six limitations. First, 2-dose and 3-dose vaccine recipients were similar in terms of most demographic and clinical characteristics but might have differed with respect to exposure risk for SARS-CoV-2 infection or risk factors for severe COVID-19. Therefore, residual or unmeasured confounding was possible. Second, VE associated with newly emergent variants, including Omicron, was not assessed. Third, VE was not assessed against SARS-CoV-2 infection or mild illness. Fourth, most 3-dose mRNA vaccine recipients were vaccinated within several weeks of enrollment and durability of protection will require future analysis. Fifth, VE associated with a fourth mRNA vaccine dose, recommended as a booster dose in immunocompromised individuals ≥5 months after dose 3, was not assessed. Finally, although medical centers in 18 states were included in the analysis, patients might not be representative of the general U.S. population.

Among adults with and without immunocompromising conditions who were eligible to receive a third dose of COVID-19 mRNA vaccine, third doses were found to increase protection beyond that of a 2-dose vaccination series for the prevention of COVID-19 hospitalization. Administration of a third COVID-19 mRNA vaccine dose as part of a primary series among immunocompromised adults, or as a booster dose among immunocompetent adults, provides improved protection against COVID-19 hospitalization.

**Summary**

**What is already known about this topic?**

For adults aged ≥18 years who received 2 doses of an mRNA COVID-19 vaccine, third doses are recommended. However, the associated benefits in preventing COVID-19 hospitalization are incompletely understood.

**What is added by this report?**

In a study of hospitalized adults, compared with receipt of 2 mRNA COVID-19 vaccine doses, receipt of a third dose increased vaccine effectiveness against hospitalization among adults without and with immunocompromising conditions, from 82% to 97% and from 69% to 88%, respectively.

**What are the implications for public health practice?**

Administration of a third COVID-19 mRNA vaccine dose as part of a primary series among immunocompromised adults, or as a booster dose among immunocompetent adults, provides improved protection against COVID-19–associated hospitalization.

## Footnotes

* Persons with immunocompromising conditions defined by the IVY Network included those with one or more of the following: active solid organ cancer (active cancer was defined as treatment for the cancer or newly diagnosed cancer in the past 6 months); active hematologic cancer (such as leukemia, lymphoma, or myeloma); HIV infection without AIDS; AIDS; congenital immunodeficiency syndrome; previous splenectomy; prior solid organ, stem cell, or bone marrow transplant; use of immunosuppressive medication; systemic lupus erythematosus; rheumatoid arthritis; psoriasis; scleroderma; or inflammatory bowel disease, including Crohn’s disease or ulcerative colitis.

† During the surveillance period, third doses were recommended ≥28 days after dose 2 if the patient had an immunocompromising condition to complete a primary series or at least 6 months after dose 2 as a booster dose if the patient did not have an immunocompromising condition. The booster dose for Moderna (0.25 mL) is one half the dose used for the primary series (0.5 mL). The third dose to complete a primary series in persons with immunocompromising conditions is the same as the first 2 doses (0.5 mL).

§ COVID-19–like illness was defined as having one or more of the following: fever, cough, shortness of breath, loss of taste, loss of smell, use of respiratory support for the acute illness, or new pulmonary findings on chest imaging consistent with pneumonia.

¶ Patients were excluded if they received a third COVID-19 mRNA vaccine dose before the FDA EUA (before August 12, 2021 for patients with immunocompromising conditions or before September 22, 2021 for patients without immunocompromising conditions [when a single booster dose of the Pfizer-BioNTech vaccine was authorized]), were admitted to the hospital before ≤7 days had elapsed since EUA authorization (before August 19, 2021 for patients with immunocompromising conditions or before September 29, 2021 for patients without immunocompromising conditions) or received a third dose before recommended during the surveillance period (<28 days after dose 2 for patients with immunocompromising conditions or <180 days after dose 2 for patients without immunocompromising conditions).

** 45 C.F.R. part 46.102(l)(2), 21 C.F.R. part 56; 42 U.S.C. Sect. 241(d); 5 U.S.C. Sect. 552a; 44 U.S.C. Sect. 3501 et seq.

†† The definition adapted from CDC definitions of moderately to severely immunocompromising conditions included having one or more of the following: active solid organ cancer (active cancer defined as treatment for the cancer or newly diagnosed cancer in the past 6 months), active hematologic cancer (such as leukemia, lymphoma, or myeloma), AIDS, congenital immunodeficiency syndrome, previous solid organ, stem cell, or bone marrow transplant, or active immunosuppressive medication use.

§§
https://www.cdc.gov/vaccines/covid-19/clinical-considerations/covid-19-vaccines-us.html
